# Comment on “Augmentation mammaplasty by superolateral thoracic flap: a case report”

**DOI:** 10.1186/s13256-022-03602-5

**Published:** 2022-10-17

**Authors:** Yanis Berkane, Nicolas Bertheuil

**Affiliations:** 1 Department of Plastic, Reconstructive and Aesthetic Surgery, Rennes University Hospital Center, Hospital Sud, University of Rennes 1, Rennes, France; 2 Vascularized Composite Allotransplantation Laboratory, Massachusetts General Hospital, Harvard Medical School, Boston, MA USA; 3 INSERM U1236, University of Rennes 1, Rennes, France; 4 SITI Laboratory, Rennes University Hospital, Rennes, France

Dear Sir,

We read with great interest the report “Augmentation mammaplasty by superolateral thoracic flap: a case report” by Lupon *et al*. [1]. We congratulate the authors for this case report and the surgical result. We are familiar with the difficulty of performing breast surgery in a post-bariatric situation, and the complexity of the volume/weight ratio in view of the poor skin tone that leads to unpredictable results.

Lupon *et al*. clearly described their superolateral thoracic flap technique, which ensures viability by conserving the external mammary vascular network and by adequate burial of the flap, allowing its harmonious integration with the breast contour. They describe the basic postoperative results: discharge on day 1, no postoperative complications, and patient satisfaction with the cosmetic result. We would like to discuss some points that the authors raise, based on our experience and previous research.

First, we agree that alternative procedures to implants should be considered for patients who have undergone massive weight loss (MWL) because the skin laxity and lack of dermal thickness leads to rapid ptosis of the reconstructed breast and the risk of secondary implant malposition [2]. However, in selected patients who have retained good-quality skin, the use of implants can be very effective, with good cosmetic results for several years [3]. Augmentation with implants remains the most predictable and suitable reconstruction of breasts following MWL, and numerous breast self-augmentation techniques with autologous tissue have been described for this population [4–7].

We described the use of lateral thoracic propeller perforator flaps buried under the mammary gland, which allows the restoration of good breast projection [6]. We proved that this technique is reproducible in nine patients, with the patients scoring the cosmetic results as 3.8 ± 0.8 out of 5. This surgery was associated with bilateral brachioplasty, as part of a total reconstruction of the upper body (an upper body lift). We believe that this approach allows a more harmonious overall silhouette. With the technique of Lupon *et al*., is possible to avoid excess skin and fat, such as that found in the axillary line (Fig. 7). However, the patient must accept the resulting scarring and the postoperative scar care required to improve it.

Moreover, Lupon *et al*. deplore the fact that their technique is difficult to associate with an inverted T-shaped scar. However, this patient population most often presents with significant breast ptosis that necessitates an inverted T scar. As we showed previously [6], self-augmentation with a classic inverted T scar can be performed if the perforating vessels of the lateral flap are skeletonized. The flap volume can be managed easily with an implant. The disadvantage is that this greatly increases the operating time, unlike the authors’ technique.

Finally, we would like to emphasize that the technique described by Hurwitz et al. [8], whose principles are rather similar to those of Lupon *et al*., benefits from hindsight due to the large number of cases described, and the expertise of this distinguished surgeon with innovative contouring surgery in MWL patients. His technique, cited by Lupon *et al*., ensures satisfactory results in terms of the projection, and is compatible with an inverted T-scar to correct the major ptosis frequently found in MWL patients. Again, integration with a full upper body lift seems preferable. Figure 1 summarizes the differences among the three variants of this technique.

**Fig. 1:**
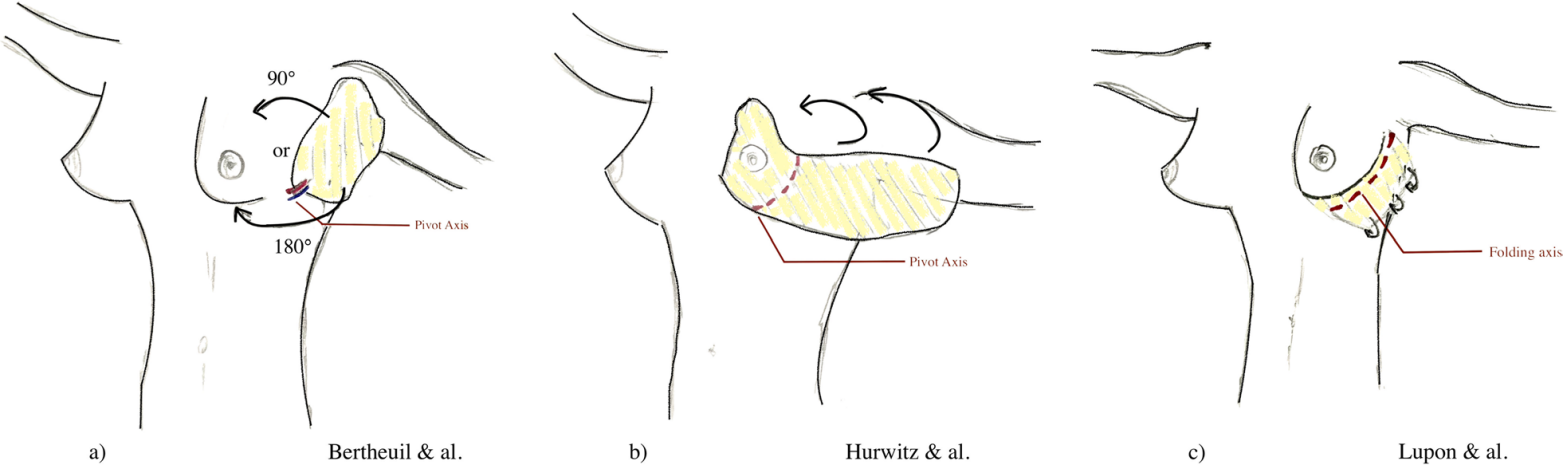
Comparison among three variations of breast auto-augmentation using a laterothoracic flap. (Berkane, 2021)

In conclusion, we agree with Lupon *et al*. that autologous breast augmentation techniques should be promoted, and we find the discovery of new techniques and modifications of existing techniques interesting. We thank Lupon *et al*. for their contribution to post-bariatric breast reconstruction, and for this interesting case report. We believe that plastic surgeons must keep innovating to improve the outcomes for MWL patients for whom surgery is not just about comfort.

## Data Availability

All the data provided in this letter is available and can be provided to the editorial board if needed.
